# Educational Responses to Students With High Abilities From the Parental Perspective

**DOI:** 10.3389/fpsyg.2019.01187

**Published:** 2019-05-24

**Authors:** Elena Rodríguez-Naveiras, María Cadenas, África Borges, Dolores Valadez

**Affiliations:** ^1^Facultad de Ciencias Sociales, Universidad Europea de Canarias, La Orotava, Spain; ^2^Facultad de Ciencias de la Salud, Universidad Europea de Canarias, La Orotava, Spain; ^3^Department of Clinical Psychology, Psychobiology and Methodology, Universidad de La Laguna, La Laguna, Spain; ^4^Centro Universitario de Ciencias de la Salud, Universidad de Guadalajara, Guadalajara, Mexico

**Keywords:** educational response, students with high abilities, parents, questionnaire, educational system

## Abstract

Students who have high abilities demand educational responses, both inside and outside of the classroom. The best type of educational strategy depends on the characteristics of the students, the school, the educational system, and the country. For this reason, the level of attention paid to high-ability students can vary across nations. To guarantee the efficacy of programs that are implemented both inside and outside of school, it is essential to offer to these students the support that they need. The students' parents are a fundamental component of this scenario. This study evaluates the educational support provided to students with high abilities from a parental perspective. This study's aim is to evaluate the opinions that parents in several countries hold about the educational responses to gifted students, as well as to compare the types of strategies implemented in these countries' respective educational systems. Parents of students with high abilities completed an *ad hoc* online questionnaire that was designed to identify the types of educational responses, the students' participation in enrichment programs (inside and outside of school), the parents' level of satisfaction with these programs, and any difficulties or problems that occurred. A mixed methodology was used, with both quantitative and qualitative survey questions. ALCESTE software was used to analyze the open-ended (qualitative) questions. This research, which was directed by the Network of Research, Intervention and Evaluation in High Intellectual Abilities, focused on three countries: Mexico, Argentina, and Spain. Descriptive analysis were carried out for analyzing the questions related with the educational support and demographic information. Qualitative analysis were used to analyze open-ended questions. The results provide evidence on which types of educational responses are being implemented in those countries, how they are being used, and whether they offer appropriate support for the needs of high-ability students.

## Introduction

Students with high intellectual abilities constitute a highly heterogeneous group, which is why there is no consensus about the characteristics that define them (López Andrada et al., 2000; Colangelo and Davis, 2003; Pérez, 2006; Matthews and Yun Dai, 2014). However, these students' cognitive functioning differs from that of their normative peer (Sastre-Riba and Domenech, 1999; Sastre-Riba, 2008). Researchers have confirmed that students with high intellectual abilities have cognitive activity that is characterized by high learning speed, good capacity for (and flexibility in) understanding and solving complex problems, high efficiency at generating new strategies to solve each problem, and intellectual precocity (Sastre-Riba and Domenech, 1999; Cross and Coleman, 2005; Van Tassel-Baska, 2013; Verche et al., 2018). According to Geake (2009), these traits can be explained by this group's greater plasticity and efficiency, which contribute to extensive attentional processes that facilitate the management of cognitive performance through working memory, flexibility, and inhibition (Sastre-Riba and Viana-Sanz, 2016; Sastre-Riba and Ortiz, 2018; Rodríguez-Naveiras, in preparation).

With regard to high-ability students' cognitive needs, three educational responses can be distinguished, according to the classification established by Southern and Jones (2004): acceleration, enrichment, and grouping. Researchers have described the characteristics of each of these strategies in depth (e.g., Colangelo and Davis, 2003; Colangelo and Assouline, 2009; Subotnik et al., 2011; Tourón, 2012; Elices et al., 2013).

Briefly, the acceleration response consists of promoting students' educational advancement at a higher speed than regulations typically allow; the enrichment response involves the implementation of activities or programs that add depth to certain aspects of the curriculum; finally, the grouping response consists of providing students with full-time or part-time education separate from their regular classrooms; or to create different groups within the classroom according to the level or ability of the students. These educational strategies have all been implemented in many countries for years. However, their effects on participants are still under investigation. Recently, with regard to the effects of participating in enrichment programs, researchers have reported positive results at the personal and academic levels (Borges et al., 2016a, 2017; Kim, 2016; Rodríguez-Naveiras and Borges, 2016; Cadenas and Borges, 2017; Jen, 2017; Jen et al., 2017; Rodríguez-Dorta et al., 2017). Similarly, researchers have identified the benefits of acceleration (Colangelo et al., 2004; Kulik, 2004; Jiménez et al., 2006; Hoogeveen, 2008; Colangelo and Assouline, 2009; Hoogeveen et al., 2012). Finally, full-time education in specialized centers also represents an adequate option, as it has produced positive results in terms of academic performance and development (Pérez, 2012; Tourón, 2012), with a greater presence outside Spain.

The types of strategies that should be carried out depend on the student, the school, the family, the educational system and, of course, the country's legislation. Choosing a strategy is a decision of vital importance, and it requires the rigor and scientific basis that research can provide.

All the countries involved on this research have current legislation that relates to students with high intellectual abilities. Spain's Organic Law (Ley Orgánica 2/2006, del 3 de mayo de Educación) (LOE)^1^, obligates school administrations to adopt measures that allow for the optimal development of these students capacities. In addition, the Span's Organic Law (Ley Orgánica 8/2013, de 9 de diciembre)^2^, relates to improving educational quality for these students; it modifies the previous law.

In Mexico, the Ministry of Education (SEP, 2006) proposed that enrichment be applied in the classroom to serve high-ability students. Subsequently, in 2013, children were allowed to attend school before the standard age or to advance by a grade level if there was reason to support such actions. In 2017, the General Education Law was modified to create a full-time education program for the gifted population.

Argentina first considered the education of children with high abilities in 1992 with the approval of the Federal Law of Education (Ministerio de Educación, 1993). Currently, the National Education Law is in force; it proposes that programs be designed for the identification, early evaluation, monitoring, and orientation of high-ability students, as well as to allow those students greater flexibility.

It is worth highlighting that, in these countries, even though there are laws that regulate the attention provided to these students, there is still a long way to go before every student receives the education they need. A previous study confirms this idea (Borges et al., 2016b) where 274 parents from different nationalities with children with high abilities completed a survey about the type of educational intervention received. Results obtained showed that the 57% of the sample responded their children did not receive any sort of educational response at school. Specifically, in Spain in the 2016–2017 school year, out of all 8,135,876 students enrolled from preschool through high school, only 27,133 (0.33% of the total enrolled) received such an intervention (Ministerio de Educación y Formación Profesional, 2017). Similarly, in Mexico in the 2017–2018 academic year, there were 14,020,204 primary-school students, but only 107,365 pupils received educational support, that is, 0.7% (SEP, 2018). These are striking figures considering that the proportion of gifted students—understood as being those with an intellectual quotient ≥130—should be approximately 2%; this proportion increases to between 10 and 20% if the focus is on talents instead of intellect (Hernández and Gutiérrez-Sánchez, 2014).

The family plays a fundamental role in the development of children with high intellectual abilities. One of the most prominent and relevant models in this framework is Brofenbrenner's (1976) ecological model (see also Bronfrenbrenner and Morris, 1998; Arranz, 2005; Bronfenbrenner, 2005). This model identifies the systems that can affect both a person's development and his or her behavior. Of special relevance is the microsystem, which is defined by a person's interaction with his or her family and close friends. Within the family context, this model distinguishes between two types of influential variables: ecological (e.g., socioeconomic status or parental age) and interactive (e.g., parental educational style). Researchers have studied how factors such as culture and socioeconomic level relate to the diagnosis of high capacities (Miller and Gentry, 2010; Castellanos Simons et al., 2015), and they have found that students from medium and medium-high socioeconomic status are more likely than those of lower status to be diagnosed with high intellectual abilities. The family context is also of great relevance in the theoretical models that seek to explain the origin and development of high intellectual capacities from a systemic perspective (Tannenbaum, 1986; Mönks, 1992; Ziegler, 2005; Ziegler and Phillipson, 2012; Gagné, 2015). However, only a few researchers have addressed the education of high-ability students from the parental perspective (Garn et al., 2010, 2012; Cadenas and Borges, 2012; Weber and Stanley, 2012; Garn and Jolly, 2015; Rodríguez-Naveiras and Borges, 2016; Borges et al., 2017; Rodríguez-Dorta et al., 2017).

This work is intended to address parents' opinions of the educational responses that their children receive. The specific objectives of the study are (1) to examine parents' understanding of educational responses; (2) to detect which types of educational responses are implemented and what parents believe about them; and (3) to identify the problems that both parents and children have in relation to the children's schools.

## Materials and Methods

### Methodology and Design

This research used a mixed methodology (Johnson and Onwuegbuzie, 2004; Denscombe, 2008). The data were collected using a cross-sectional design based on a survey composed of open-ended questions that allow for qualitative analysis.

### Participants

The participants in this research comprised 243 Spanish-speaking parents. Most of the participants were from Spain (52.7%), and the remainder were from Mexico (35.4%) or Argentina (11.9%). The mean (standard deviation) age of the participants was 41.42 (5.49) years, and 87.2% of the parents were female. Regarding the participants' educational level, 44.9% had an undergraduate university degree but not a graduate degree, and another 25.5% went to High School. Finally, 27.2% of the participants had one child who had been diagnosed with high abilities, and 61.3% had two such children. The data for gender, nationality, level of education, and number of children diagnosed are shown in Table 1.

**Table 1:** Description of the participants.

	***N***	**%**
Gender		
Male	31	12.8
Female	212	87.2
Country		
Spain	128	52.7
Mexico	86	35.4
Argentina	29	11.9
Highest level Of education completed		
Primary school	11	4.5
High school	62	25.5
Undergraduate degree	109	44.9
Master's degree	45	18.5
PhD degree	16	6.6
Number of children diagnosed		
1	66	27.2
2	149	61.3
3	24	9.9
4	3	1.2
5	1	0.4

*%, percentage*.

### Instruments

An *ad hoc* online questionnaire was designed to collect the data; it consists of 68 open-ended and closed-ended questions on the following aspects: the diagnosis, the type of educational response, the children's participation in intra- and extracurricular programs, the degree of satisfaction with the intervention programs, and the difficulties encountered. For this study, 16 of the 68 total items were selected for analysis. The complete version of the survey in Spanish and the translation of each item into English is presented in Table 2. The quantitative and qualitative items analyzed are presented in Table 3.

**Table 2:** Survey items.

**Dimension**	**Spanish survey**	**English survey**
Identification variables	Edad	Age
	Sexo	Gender
	País	Country
	Nivel de estudio	Educational level
	Profesión/ocupación	Profession/Occupation
	Número de hijos/as	Number of children
Definition	¿Qué entiende usted por alta capacidad/superdotación/talento?	What do you understand by high capacity/giftedness/talent?
Diagnosis	¿Se ha diagnosticado a alguno de sus hijos con alguna de estas etiquetas (señalar la que se le haya dado)? Altas capacidades Superdotación Sobredotación Alumno/a sobresaliente Talento No Otro	Have any of your children been diagnosed with any of these labels (indicate which of them)? High capacities Giftedness Outstanding student Talented None Other
	1. ¿Cómo considera usted que debería hacerse el diagnóstico de altas capacidades/ superdotación/talento?	1. How do you consider the diagnosis of high abilities/giftedness/talent should be made?
	2. ¿A cuántos de sus hijos se ha diagnosticado de alta capacidad/superdotación/talento?	2. How many of your children have been diagnosed with high ability/giftedness/talent?
Educational response	3. ¿Considera que debería recibir una atención dentro de la escuela acorde a sus capacidades? Si No	3. Do you think you should receive care within the school according to your abilities? Yes No
	4. En caso afirmativo, ¿de qué tipo?	4. If the answer is yes, please mention what kind
Diagnosis	5. Edad del primer hijo/a diagnosticado	5. Age of the first diagnosed child
	6. Sexo del primer hijo/a diagnosticado Varón Mujer	6. Gender of the first diagnosed child Male Female
	7. ¿Qué profesional (incluir la titulación) realizó el diagnóstico?	7. What professional (please include the degree) did the diagnosis?
	8. ¿Qué opinión le merece el diagnóstico realizado a su hijo/a?	8. What is your opinion of the diagnosis made to your child?
	9. En cuanto al tiempo transcurrido desde la detección (cuando alguien consideró que podía ser de altas capacidades, hasta que se le pasaron las pruebas para determinar el diagnóstico), usted está: 1. Nada de acuerdo 2. En desacuerdo 3. Ni de acuerdo ni en desacuerdo 4. De acuerdo 5. Totalmente de acuerdo	9. Regarding the time elapsed since the detection/diagnosis (when someone considered that he/she could be of high abilities, until he/she passed the tests to determine the diagnosis), what are your feelings: 1. Totally disagree 2. Disagree 3. Neither agree nor disagree 4. Agree 5. Totally agree
	10. En cuanto a la capacidad profesional de quienes realizaron el diagnóstico, usted está: 1. Nada de acuerdo 2. En desacuerdo 3. Ni de acuerdo ni en desacuerdo 4. De acuerdo 5. Totalmente de acuerdo	10. Regarding the professional capacity of those who carried out the diagnosis, what are your feelings: 1. Totally disagree 2. Disagree 3. Neither agree nor disagree 4. Agree 5. Totally agree
	11. En cuanto a las pruebas pasadas para diagnosticar las altas capacidades, usted está: 1. Nada de acuerdo 2. En desacuerdo 3. Ni de acuerdo ni en desacuerdo 4. De acuerdo 5. Totalmente de acuerdo	11. As for the tests and exams to diagnose the high capacities, you feel? 1. Totally disagree 2. Disagree 3. Neither agree nor disagree 4. Agree 5. Totally agree
	12. En cuanto a la claridad del informe del diagnóstico, usted está: 1. Nada de acuerdo 2. En desacuerdo 3. Ni de acuerdo ni en desacuerdo 4. De acuerdo 5. Totalmente de acuerdo	12. Regarding the clarity of the diagnosis report, you feel: 1. Totally disagree 2. Disagree 3. Neither agree nor disagree 4. Agree 5. Totally agree
Parent's problems with the school	14. ¿Ha tenido usted problemas con la escuela referidos a su hijo/a? Si No	14. Have you had problems with the school regarding your child? Yes No
	15. ¿Qué tipo de problema?	15. What type of problems?
	16. ¿Con quién? Equipo de orientación del centro educativo (o equipo de psicopedadagogía USAER) Dirección Profesorado Tutor/a Compañeros/as Otros progenitores Otro	16. With whom? Orientation team of the educational center (or USAER psychopedagogical team) Principal Faculty Tutor Companions Other parents Other
Children's problems at school	17. ¿Ha tenido su hijo/a problemas con la escuela? Si No	17. Has your child had problems with school? Yes No
	18. ¿Qué tipo de problema?	18. What type of problems?
	19. ¿Con quién? Servicio de orientación Dirección Profesorado Tutor/a Compañeros/as Otros progenitores Otro	19. With whom? Orientation team of the educational center (or USAER psychopedagogical team) Principal Faculty Tutor Companions Other parents Other
Educational response within the school	20.¿Ha recibido una respuesta educativa dentro de la escuela acorde a sus necesidades? Si No	20. Have you received an educational response within the school according to your needs? Yes No
	21. ¿En qué consiste la respuesta educativa que se le da en la escuela? Aceleración Agrupamiento total (todas las clases las recibe con alumnado exclusivamente de altas capacidades) Agrupamiento parcial (algunas clases las recibe con alumnado exclusivamente de altas capacidades) Enriquecimiento Adaptación curricular Otro	21. What is the educational response that is given in school? Acceleration Total grouping (all classes receive them with exclusively high-ability students) Partial grouping (some classes receive them with exclusively high-ability students) Enrichment Curricular adaptation Other
	22. ¿Cuánto tiempo tardó en tener una respuesta educativa (especificar meses o años)?	22. How long did it take to have an educational response (specify months or years)?
	23. ¿Cuánto tiempo hace que comenzó la respuesta educativa que ha recibido su hijo/a? (Especificar meses o años)	23. How long ago did the educational response your child received begin? (Specify months or years)
	24. ¿Lo sigue recibiendo aún?	24. Do you still receive it?
	25. ¿Por qué?	25. Why?
	26. Fecha de finalización (mes y año)	26.Date of completion (month and year)
	27. En cuanto a la temporalización (cuándo se desarrolla) 1. Nada satisfecho 2. Insatisfecho 3. Ni satisfecho ni insatisfecho 4. Satisfecho 5. Totalmente satisfecho	27. Regarding timing (when it develops), you feel: 1. Not satisfied 2. Dissatisfied 3. Neither satisfied nor dissatisfied 4. Satisfied 5. Completely satisfied
	28. En cuanto al profesorado que lo implementa 1. Nada satisfecho 2. Insatisfecho 3. Ni satisfecho ni insatisfecho 4. Satisfecho 5. Totalmente satisfecho	28. In terms of the faculty that implements it, you feel: 1. Not satisfied 2. Dissatisfied 3. Neither satisfied nor dissatisfied 4. Satisfied 5. Completely satisfied
	29. En cuanto a los contenidos que recibe 1. Nada satisfecho 2. Insatisfecho 3. Ni satisfecho ni insatisfecho 4. Satisfecho 5. Totalmente satisfecho	29. Regarding the contents received, you feel: 1. Not satisfied 2. Dissatisfied 3. Neither satisfied nor dissatisfied 4. Satisfied 5. Completely satisfied
	30. ¿El programa le ayuda a obtener mejores calificaciones escolares? Si No	30. Does the program help you get better grades? Yes No
Educational response out of the school	31. ¿Participa (o ha participado) en algún programa extra escolar? Si No	31. Do you participate (or have you participated) in any extra-curricular program? Yes No
	32. Tipo de programa Cognitivo Socioafectivo Ambos No sabe	32. Type of extra-curricular program: Cognitive Socio-affective Both Do not know
	33.Nombre del programa	33.Program name:
	34. ¿Quién lo organiza?	34. Who organizes it?
	35. ¿Cuánto tiempo hace que comenzó a asistir al programa extraescolar (meses o años)	35. How long since you started attending the after-school program (months or years)?
	36. ¿Aún participa? Sí No	36.Are you still participating? Yes No
	37. ¿Por qué?	37. Why?
	38. Fecha de finalización (mes y año)	38. End date (month and year)
	39. En cuanto a la temporalización (cuándo se desarrolla): 1. Nada satisfecho 2. Insatisfecho 3. Ni satisfecho ni insatisfecho 4. Satisfecho 5. Totalmente satisfecho	39. Regarding timing (when it develops): 1. Not satisfied 2. Dissatisfied 3. Neither satisfied nor dissatisfied 4. Satisfied 5. Completely satisfied
	40. En cuanto al profesorado que lo implementa 1. Nada satisfecho 2. Insatisfecho 3. Ni satisfecho ni insatisfecho 4. Satisfecho 5. Totalmente satisfecho	40. Regarding the teaching staff that implements it 1. Not satisfied 2. Dissatisfied 3. Neither satisfied nor dissatisfied 4. Satisfied 5. Completely satisfied
	41. En cuanto a los contenidos que recibe 1. Nada satisfecho 2. Insatisfecho 3. Ni satisfecho ni insatisfecho 4. Satisfecho 5. Totalmente satisfecho	41. In terms of the content received 1. Not satisfied 2. Dissatisfied 3. Neither satisfied nor dissatisfied 4. Satisfied 5. Completely satisfied
	42. ¿El programa le ayuda a obtener mejores calificaciones escolares? Si No	42. Does the program help you get better grades? Yes No
	43. ¿Lo recomendaría a otras familias? Si No	43. Would you recommend it to other families? Yes No
	44. ¿Por qué?	44. Why?
	45. ¿El programa tiene también un programa para progenitores? Si No	45. Does the program also provide a parent program? Yes No
	46. En cuanto a la temporalización (cuándo se desarrolla) 1. Nada satisfecho 2. Insatisfecho 3. Ni satisfecho ni insatisfecho 4. Satisfecho 5. Totalmente satisfecho	46. Regarding timing (When it develops), you feel: 1. Not satisfied 2. Dissatisfied 3. Neither satisfied nor dissatisfied 4. Satisfied 5. Completely satisfied
	47. En cuanto al profesorado que lo implementa 1. Nada satisfecho 2. Insatisfecho 3. Ni satisfecho ni insatisfecho 4. Satisfecho 5. Totalmente satisfecho	47. Regarding the faculty that implements it. You feel: 1. Not satisfied 2. Dissatisfied 3. Neither satisfied nor dissatisfied 4. Satisfied 5. Completely satisfied
	48. En cuanto a los contenidos que recibe 1. Nada satisfecho 2. Insatisfecho 3. Ni satisfecho ni insatisfecho 4. Satisfecho 5. Totalmente satisfecho	48.As for the content received, you feel: 1. Not satisfied 2. Dissatisfied 3. Neither satisfied nor dissatisfied 4. Satisfied 5. Completely satisfied
	49. En cuanto a la aplicación del programa en su labor como progenitor 1. Nada satisfecho 2. Insatisfecho 3. Ni satisfecho ni insatisfecho 4. Satisfecho 5. Totalmente satisfecho	49.Regarding the application of the program work as a parent, you feel: 1. Not satisfied 2. Dissatisfied 3. Neither satisfied nor dissatisfied 4. Satisfied 5. Completely satisfied
	50. ¿Participa en otro programa extraescolar para altas capacidades? Si No	50. Do you participate in another after-school program for high abilities? Yes No
	51. Tipo de programa Cognitivo Socioafectivo Ambos No se sabe	51. Type of program? Cognitive Socio-Affective Both Do not know
	52. Nombre del programa	52. Program name:
	53. ¿Quién lo organiza?	53. Who organizes it?
	54. ¿Cuánto tiempo hace que comenzó a asistir al programa extraescolar (meses o años)	54. How long ago did you start attending the after-school program (months or years)?
	55. ¿Aún participa?	55. Are you still participating?
	56. ¿Por qué?	56. Why?
	57. Fecha de finalización (mes y año)	57. End date (month and year)
	58. En cuanto a la temporalización (cuándo se desarrolla) 1. Nada satisfecho 2. Insatisfecho 3. Ni satisfecho ni insatisfecho 4. Satisfecho 5. Totalmente satisfecho	58. Regarding timing (when it develops), you feel: 1. Not satisfied 2. Dissatisfied 3. Neither satisfied nor dissatisfied 4. Satisfied 5. Completely satisfied
	59. En cuanto al profesorado que lo implementa 1. Nada satisfecho 2. Insatisfecho 3. Ni satisfecho ni insatisfecho 4. Satisfecho 5. Totalmente satisfecho	59. Regarding the faculty that implements it, you feel: 1. Not satisfied 2. Dissatisfied 3. Neither satisfied nor dissatisfied 4. Satisfied 5. Completely satisfied
	60. En cuanto a los contenidos que recibe 1. Nada satisfecho 2. Insatisfecho 3. Ni satisfecho ni insatisfecho 4. Satisfecho 5. Totalmente satisfecho	60. Regarding the contents received, you feel: 1. Not satisfied 2. Dissatisfied 3. Neither satisfied nor dissatisfied 4. Satisfied 5. Completely satisfied
	61. ¿El programa le ayuda a obtener mejores calificaciones escolares? Si No	61. Does the program help you get better grades? Yes No
	62. ¿Lo recomendaría a otras familias? Si No	62. Would you recommend it to other families? Yes No
	63. ¿Por qué?	63. Why?
	64. ¿El programa tiene también una sección para padres? Si No	64.Does the program also have a section for parents? Yes No
	65. En cuanto a la temporalización (cuándo se desarrolla) 1. Nada satisfecho 2. Insatisfecho 3. Ni satisfecho ni insatisfecho 4. Satisfecho 5. Totalmente satisfecho	65. Regarding timing (when it develops), you feel: 1. Not satisfied 2. Dissatisfied 3. Neither satisfied nor dissatisfied 4. Satisfied 5. Completely satisfied
	66. En cuanto al profesorado que lo implementa 1. Nada satisfecho 2. Insatisfecho 3. Ni satisfecho ni insatisfecho 4. Satisfecho 5. Totalmente satisfecho	66. Regarding the teaching staff that implements it, you feel: 1. Not satisfied 2. Dissatisfied 3. Neither satisfied nor dissatisfied 4. Satisfied 5. Completely satisfied
	67. En cuanto a los contenidos que recibe 1. Nada satisfecho 2. Insatisfecho 3. Ni satisfecho ni insatisfecho 4. Satisfecho 5. Totalmente satisfecho	67. Regarding the contents received, you feel: 1. Not satisfied 2. Dissatisfied 3. Neither satisfied nor dissatisfied 4. Satisfied 5. Completely satisfied
	68. En cuanto a la aplicación del programa en su labor como progenitor 1. Nada satisfecho 2. Insatisfecho 3. Ni satisfecho ni insatisfecho 4. Satisfecho 5. Totalmente satisfecho	68. Regarding the application of the program work as a parent you feel: 1. Not satisfied 2. Dissatisfied 3. Neither satisfied nor dissatisfied 4. Satisfied 5. Completely satisfied

**Table 3:** List of selected quantitative and qualitative items.

**Number**	**Question and possible responses**
**QUANTITATIVE QUESTIONS**	
Filter question 3	Have any of your children been diagnosed with any of these labels (indicate which of them)? Answer: High capacities/ Giftedness /Outstanding student/Talented/None/Other.
3	Do you think you should receive care within the school according to your abilities? Answer: yes/no
14	Have you had problems with the school regarding your child? Answer: yes/no
17	Has your child had problems with school? Answer: yes/no/no answer
20	Have you received an educational response within the school according to your needs? Answer: yes/no/no answer
21	What is the educational response that is given in school? Answer: Acceleration/Total grouping (all classes receive them with exclusively high-ability students)/ Partial grouping (some classes receive them with exclusively high-ability students)/ Enrichment/ Curricular adaptation/Other
22	How long did it take to have an educational response (specify months or years)?
27-29	What is your degree of satisfaction with the support that your child receives (or has received) with regard to each of the following? timing faculty content Answer: Not satisfied/Dissatisfied/Neither satisfied nor dissatisfied/Satisfied/ Completely satisfied
30	Does the program help you get better grades? Answer: yes/no/no answer
31	Do you participate (or have you participated) in any extra-curricular program? Answer: yes/no/no answer
32	Type of extra-curricular program: Answer: Cognitive/Socio-Affective/Both /Do not know
**Number**	**Question**
**QUALITATIVE QUESTIONS**	
4	Do you think you should receive care within the school according to your abilities? If the answer is yes, please mention what kind
15	What kind of problems? (parent)
17	What kind of problems? (child)

### Procedure

The University of La Laguna's Ethics Committee of Research and Animal Welfare has approved this research (registration number CEIBA2018-0300). In addition, the Network for Research, Intervention and Evaluation in High Intellectual Capabilities-REINEVA (http://reineva.gtisd.net/) participated in the dissemination of the questionnaire; this group's objective is to integrate specialists who are interested in studying high-ability children. The data-collection process began in May 2018 and ended in October of the same year.

The questionnaire was answered by adults over the age of 18 who are parents of children with high capacities. The questionnaire was filled in online; by answering it participants gave informed consent. Additionally, the aim of the research was included before completing the survey. This questionnaire is totally anonymous and no personal information was requested. We asked the parents' opinion of the educational response to their diagnosed children.

Once the data were collected, the participants were selected based on the filter question, “Which of the following labels has the school applied to your child?” Only the parents who listed a label of gifted on this question were considered.

After the data collection the analysis previously described were carried out with the answers of the participants in their mother tongue (Spanish). After the analysis, the results were translated into English.

### Data Analysis

The quantitative analysis was carried out using SPSS software (version 23). To determine the relationship between the variables and the participants' country, Cramer's *V* coefficient was also calculated.

The qualitative analysis was performed using ALCESTE software (Reinert, 2003). Use of this program has been widely described (Illia et al., 2014; Courtinat-Camps et al., 2017; Zambrano, 2017).

ALCESTE is a software developed by Reinert in 1986 which uses statistical procedures to perform textual analysis to extract the essential information from a text. The aim is to identify and quantify the strongest structures from a text. The program uses a statistical procedure which clusters groups of text through chi square. Additionally, it extracts a correspondence factorial analysis.

This methodology focusses in the statistical distribution of series of words which composes the statements of a text, taking into account the co-ocurrence, that is, the simultaneous presence of several words (nouns, adjectives and verbs), removing the analysis of prepositions or conjunctions, etc. The objective is to differentiate the most significant lexical worlds (main groups of words which have a repetitive pattern throughout the text and with a similar meaning).

The unit of analysis is the Unit of Elementary Context (UEC) which corresponds with the main idea of the sentence with 8 or 20 words by UEC (De Alba, 2004). This procedure permits to discover relations between lexical universes. The co-ocurrence is by association of proximity.

One of the advantages of this software is that it is not a person who codifies, like in other programmes as Atlas-ti. On the contrary, it is the software which establishes connections using statistical procedures (Bauer, 2003). The main advantages of ALCESTE (Illia et al., 2014) are: a researcher does not impose his/her interpretation of which part of the text is different from or similar to others. For this reason, human bias is controlled. It is really useful when it is necessary to analyze large passages of text. This procedure has been used in different topics like the study of diagnosis of Down Syndrome (Torres and Maia, 2009), in health psychology (Parrello and Osorio-Guzmán, 2013) and, specifically in gifted education (Villate and De Leonardis, 2012; Courtinat-Camps et al., 2017).

## Results

To answer the first objective of this research, two questions have been posed: if they consider that they should have a specific educational response and, if so, what they think it should be. In response to the first question, the parents mostly consider that these students should receive a specific educational response, since only 4 of the 244 interviewees (1.6% of the total) disagree.

Once the parents' opinions on the educational response that their children of high abilities have been analyzed, it was asked if their children had received an educational response according to their needs. Only 60 responded affirmatively, representing 24.7% of the sample interviewed. There is no significant association between educational response and country of origin (Cramer's *V* = 0.36, *p* = 0.857). The distribution of educational response according to the countries studied is presented in Table 4.

**Table 4:** Educational responses to high-capacity students, by country.

**Country**	**Educational response**	
	**Yes**	**No**
Spain	98	30
Mexico	63	23
Argentina	22	7

To know the opinion that parents have regarding the educational response that their children should receive, we carried out two analyses using ALCESTE, one for which their children receive specific training and those that do not.

**Figure 1:**
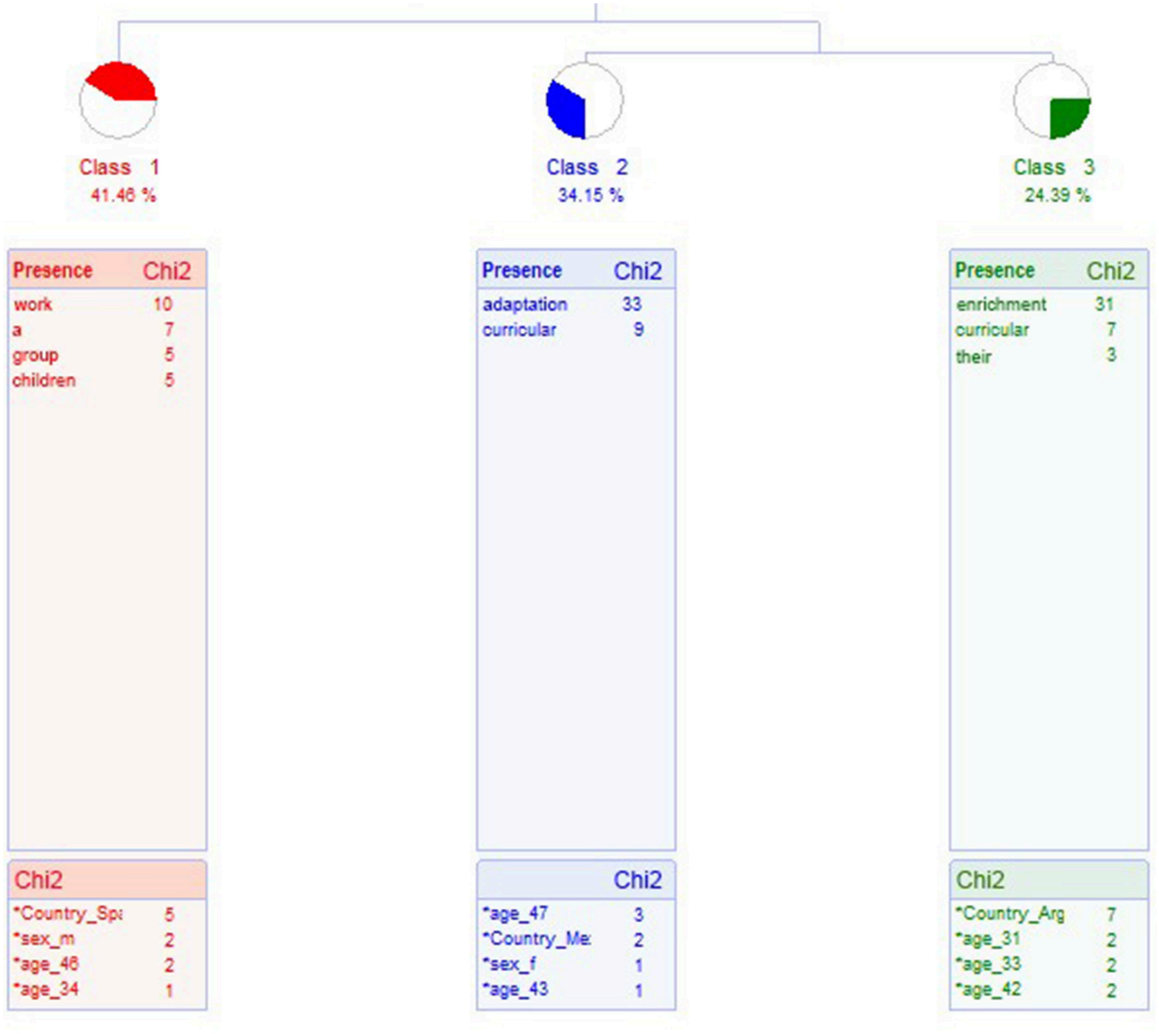
Dendrogram about what may be the educational response for high ability. Parents whose children receive an educational response for their talent.

In the first case, the analysis yields three classes, which classify 41 Elementary Contextual Units (ECUs), explaining 65% of the corpus. A dendrogram is shown in Figure 1. There are two different types of class: class 1, more unspecific, and class 2 and 3, related with common educational interventions in the education of students with high intellectual abilities.

The class that groups the most ECUs is 1, with 17 ECUs, which represents 41.46% of the ECUs, called Way of Working. The most representative word is *work*. Examples:

La forma de trabajar para estos niños es importante. no puede ser algo repetitivo, se aburre. debiera tener motivación para centrarse en el trabajo

*The way of working for these children is important. it cannot be repetitive, he/she gets bored. should be motivated to focus on work* (chi^2^ = 9)

importante integrarlo en trabajo en grupos, que pueda ver que otras personas les pueden aportar cosas

*important to integrate him/ her in working groups, that he/she can see that other people can contribute* (chi^2^ = 9)

Class 2 groups 14 ECUs, which represents 34.15% of them. It is called Curricular Adaptation and the most representative word is *adaptation*. Examples of phrases are:

todo esto aparte de la flexibilizacion y la adaptación curricular

*all this besides flexibilization and the curricular adaptation* (chi^2^ = 6)

adaptación curricular

*curricular adaptation* (chi^2^ = 6)

Class 3 groups 10 ECUs, which represents 24.39% of them. It is called Curricular Enrichment and the most representative word is *enrichment*. Examples of ECUs are:

enriquecimiento curricular y flexibilizacion en areas concretas

*curricular enrichment and flexibilization in concrete areas* (chi^2^ = 3)

enriquecimiento curricular

*curricular enrichment* (chi^2^ = 3)

Parents were asked about the type of educational response received by their children, the time elapsed between the diagnostic and the educational response, as well as the assessment made by both the teachers who implement the programs and the content they receive. The results by countries, as well as the relationship between the variables analyzed, are presented in Table 5.

**Table 5:** Characteristics of the educational response.

**Country**					
	**Spain**	**Mexico**	**Argentina**	**Cramer's** ***V***	***p***
**TYPE OF EDUCATIONAL RESPONSE**					
Acceleration	4	2	0	0.224	0.419
Enrichment	9	6	0		
Full gifted education	2	1	0		
Several	15	14	7		
**TIME REQUIRED TO RECEIVE AN EDUCATIONAL RESPONSE**					
Less than 1 month	16	6	4	0.211	0.498
Up to 3 months	3	4	1		
3 to 6 months	2	3	1		
More than 6 months	9	10	1		
**EVALUATIONS OF THE TEACHERS WHO IMPLEMENT THE**					
**EDUCATIONAL RESPONSES**					
Strongly disagree	4	1	0	0.177	0.876
Disagree	1	1	0		
Neither agree nor disagree	5	6	2		
Agree	14	9	4		
Strongly agree	6	6	1		
**ASSESSMENTS OF THE CONTENT OF THE EDUCATIONAL RESPONSES**					
Strongly disagree	3	0	0	0.315	0.157
Disagree	1	3	0		
Neither agree nor disagree	8	2	3		
Agree	10	14	3		
Strongly agree	8	4	1		

### Educational Response

**Figure 2:**
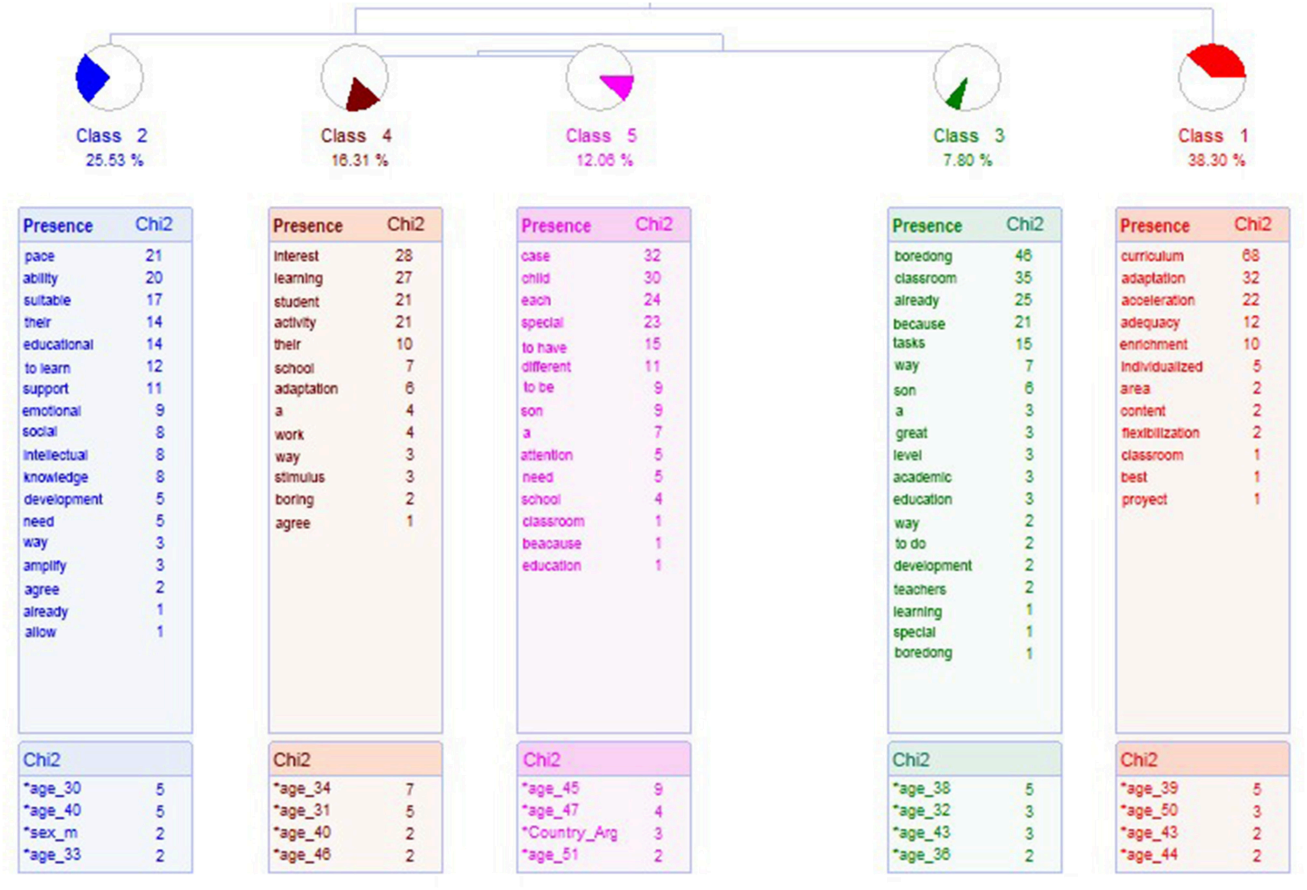
Dendrogram about what may be the educational response for high ability. Parents whose children do not receive an educational response for their talent.

In the case of parents whose children do not receive an educational response, the analysis of ALCESTE yields five classes, which classifies 141 ECUs, which constitutes 76% of the corpus (see the dendrogram in Figure 2). There is a first axis with class 1, which is related specifically to class 2, with class 3, which links with classes 4 and 5.

Class 1 groups 54 ECUs, which explain 38.30 % of the ECUs. The most representative word is curricular, and is called Traditional Educational Response in Talent. Examples of this class are the following phrases:

adaptacion curricular y/o aceleracion de curso.

*curricular adaptation and/or acceleration of year (chi*^2^ = *11)*

adaptacion curricular, enriquecimiento.

*curricular adaptation, enrichment (chi*^2^ = *11)*

Class 2, Special Pace, groups 17 ECUs, which represents 12.88% of the ECUs. The most representative word is *Pace*, gathering phrases of which the following are examples:

necesitaría otra forma de aprender y poder relacionarse con sus iguales intelectuales además de ampliar materia

*would need another way of learning and being able to relate to their intellectual equals in addition to expanding subject* (chi^2^ = 10)

poder crecer intelectualmente a su ritmo

*to be able to grow intellectually at his/her own pace* (chi^2^ = 8)

Class 3 groups 15 ECUs, and represents 7.8 % of them. It is called Boredom and the most representative word is *boredom*. Examples of phrases are indicated below.

adaptar las tareas, ya que el niño/a se puede llegar a aburrir en clase.

*adapt the tasks, since the child can get bored in class (chi*^2^ = *25)*

si ya sabe hacer algo explicarle otra cosa aun no se haya dado todavía. mayormente porque se aburren (Individu n° 48 ^*^sex_f ^*^age_43 ^*^Country_Spain)

*If he/she already knows how to do something, explain something else that has not happened yet. mostly because they get bored (chi*^2^ = *25)*

Class 4, Encourage Interest, groups 23 ECUs, which represents 16.31 % of the ECUs. The most representative word is *interes*t. Phrases that are collected in this class are presented below.

un enriquecimiento suficiente para q el alumno mantenga su interés por ir a la escuela.

*enough enrichment for the student to maintain interest in going to school* (chi^2^ = 15).

involucrándole en un estilo de aprendizaje que estimule su interés.

*involving him or her in a learning style that stimulates their interest* (chi^2^ = 15).

Class 5, Special Needs, include 17 ECUs, representing 12.06% of the corpus. The most representative phrase is *case*. Below are phrases belonging to this class.

cada niño tiene unas necesidades diferentes. adaptarse a ellas.

*each child has different needs. adapt to them* (chi^2^ = 18)

aquella que necesite cada niño, no todos son iguales, ni necesitan la misma atención.

*the one that each child needs, not all are equal, nor need the same attention* (chi^2^ = 18).

### Out of School Educational Response

Other educational response is implemented out of school. In terms of attendance at out of school programs, more than half have received this type of intervention (58%, 141 of the participants). In 30% of the cases, they combine the enrichment programs within the classroom without the school programs as educational response, while 11.93% only receive intra-school programs. It should be noted that 12.75% of those interviewed said they did not receive any type of educational response.

With regard to participation in out of school programs analyzed by the three participating countries (see Table 6), there is a significant relationship (Cramer's V = 0.248, *p* = 0.001), since in Spain and Mexico it is more frequent to participate in these programs, unlike in Argentina.

**Table 6:** Participation in out-of-school programs, by country.

**Country**	**Out-the-school programs**	
	**No**	**Yes**
Spain	57	71
Mexico	25	61
Argentina	20	9

### Parents' Problems With the School

Seventy percentage of the parents have had problems with the school, with no relationship between this variable and the country of origin: (Cramer's = 0.133, *p* = 0.117). The results by country are shown in the Table 7.

**Table 7:** Problems that parents had with schools, by country.

**Country**	**Problems of parents with the school**	
	**No**	**Yes**
Spain	40	88
Mexico	29	57
Argentina	4	25

The problems with the school have occurred even when there has been an educative response: of the 170 parents who reported having problems with the school (69.67%), 26 of them received an educational response (10.66%).

Answering about with whom or who have had problems, most often it has been with several members of the school. The relationship between who has had problems and country is significant Cramer's V = 0.215, *p* = 0.047, since Spaniards are the ones who present a higher frequency of problems with several members of the school (see Table 8).

**Table 8:** Members of the school community with whom the parents had problems.

**Country**	**Problems**				
	**1**	**2**	**3**	**4**	**5**
Spain	2	2	12	72	0
Mexico	1	1	16	37	2
Argentina	1	3	3	17	1

*1: Educational and vocational services; 2: Principal; 3: Teacher or tutor; 4: several; 5: peers/other parents*.

**Figure 3:**
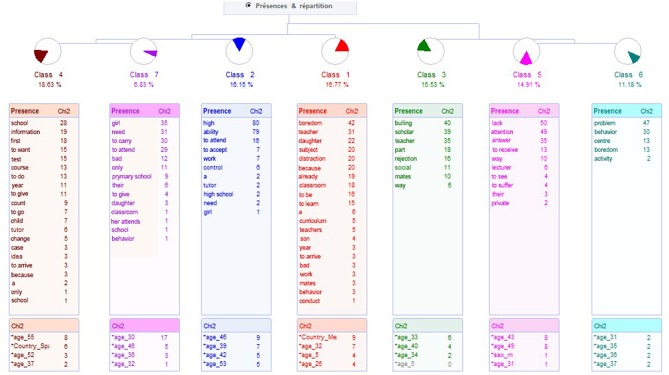
Dendrogram about parents' problems with the school.

To know the type of problems, an open question was carried out, analyzed with ALCESTE. It resulted in 7 classes, classifying 78% of the ECUs, which supposes 161 ECUs. A first axis is given, which links classes 1, 2 and this one with 4 and 7. Contents are related with problems with the school. The second axis relates class 3, which at the same time links to 5 and 6, to relate problems referred to the student. The dendrogram is presented in Figure 3.

Class 1 groups 27 units, corresponding to 16.77% of ECUs, being the most representative word *boredom*. It's called Boredom at School. Examples of phrases are:

se aburre cuando ven un tema conocido no presta atención y cuando pasan a otro el ya está distraído, le parece que todo va lento (Individu n° 41 ^*^sex_f ^*^age_34 ^*^Country_Mexico)

*he gets bored when they see a known topic he does not pay attention and when they pass to another he is already distracted, he thinks everything goes very slowly* (chi^2^ = 15)

mi hijo se aburria mucho, s portaba mal en clase, y la culpa era nuestra

*my son got bored, he behaved badly in class, and it was our fault* (chi^2^ = 12)

Class 2 groups 26 UEC, representing 16.15% of the total of ECUs. It is called Lack of Attention to High Capacities, being the most representative word is *High*. Examples of phrases are presented below.

no atienden las altas capacidades (Individu n° 43 ^*^sex_f ^*^age_42 ^*^Country_Spain)

Unité textuelle n°19 de Khi2 = 17

they don't take care of high abilities (chi^2^ = 25)

no lo consideran alta capacidad.

*they do not consider it high capacity* (chi^2^ = 17)

From class 2 comes an axis which connects class 4 and 7. Class 4 groups 30 ECUs, representing 18.63 of the total ECUs, resulting in the highest weight. It is denominated Lack of Interest on the Part of the School, being the most representative word is *School*, being examples of phrases the ones that are shown next.

baja disposición de una tutora. no se dio cuenta de las habilidades del niño (Individu n° 28 ^*^sex_f ^*^age_35 ^*^Country_Spain)

Unité textuelle n°17 de Khi2 = 16

en la primera escuela omitir el informe privado y no entregarlo a su debido tiempo en la segunda, dar por válidas las pruebas privadas y no intervenir porque el niño va bien y no da problemas.

*low disposition of his tutor. he did not realize the child's abilities* (chi^2^ = 20).

*in the first school omit the private report and not deliver it in due time in the second, validate the private tests and not intervene because the child is doing well and does not cause problems* (chi^2^ = 16).

Class 7, called Lack of Attention to Student Needs. Groups 11 ECUs, that is, 6.83 %. The most representative word is *Girl*. Examples of phrases are presented below.

solo en primer y segundo grado atendieron sus necesidades (Individu n° 105 ^*^sex_f ^*^age_32 ^*^Country_Argentina)

Unité textuelle n°8 de Khi2 = 26

no atendían al niño según sus necesidades y derivo en una mala conducta que le llevo a un principio de depresión.

only in the first and second grades did they meet their needs

*They did not take care of the child according to their needs and it led to a bad behavior that led to a beginning of depression*.

The axis that starts from class 3 connects with classes 5 and 6 and 7. Class 3 is called Bullying, group 25 UEC, which represents 15.53%. The most representative word is *Bullying*. Examples of phrases are shown in the following paragraphs.

acoso escolar por parte del alumnado. inatención y rechazo por parte del profesorado (Individu n° 73 ^*^sex_f ^*^age_44 ^*^Country_Spain)

Unité textuelle n°5 de Khi2 = 29

acoso por parte de compañeros, profesores y padres.

*bullying by students. Inattention and rejection by teachers* (chi^2^ = 36)

*bullying by mates, teachers and parents* (chi^2^ = 29)

The last axis is formed by classes 5 and 6. Class 5, Lack of Attention, groups more UEC, 24, which represents 14.91% of them. The most representative word is *Lack*. The sentence examples are shown below.

no he recibido atención (Individu n° 1 ^*^sex_f ^*^age_50 ^*^Country_Mexico)

Unité textuelle n°84 de Khi2 = 18

falta de respuesta y falta de rapidez.

*I have not received attention* (chi^2^ = 18)

*lack of response and lack of speed* (chi^2^ = 18)

Class 6 meets 18 ECUs, named Behaviors problems, represent 11.18% of the corpus, with the most representative word being *Problem*. Below are two examples of phrases of this class.

aburrimiento y problemas de conducta.

problemas con el centro, no vemos que avance adecuadamente.

*boredom and behavior problems* (chi^2^ = 30).

*problems with the center, we do not see-that he/she progresses properly* (chi^2^ = 20).

### Children's Problems With the School

Regarding whether their children have had problems with school, 184 (75.7%) of the participants respond affirmatively. Analyzing this response by countries, it is observed that the relationship is significant: Cramer's V = 0.277, *p* = 0.001, the frequency in Mexico of problems with parents and peers being higher and in Spain with several members of the school. Results are showed in Table 9.

**Table 9:** Children's problems with the school.

**Country**	**Problems**				
	**1**	**2**	**3**	**4**	**5**
Spain	4	2	15	73	1
Mexico	2	3	17	31	10
Argentina	1	3	1	19	1

*1: Educational and vocational services; 2: Principal; 3: Teacher or tutor; 4: several; 5: peers/other parents*.

**Figure 4:**
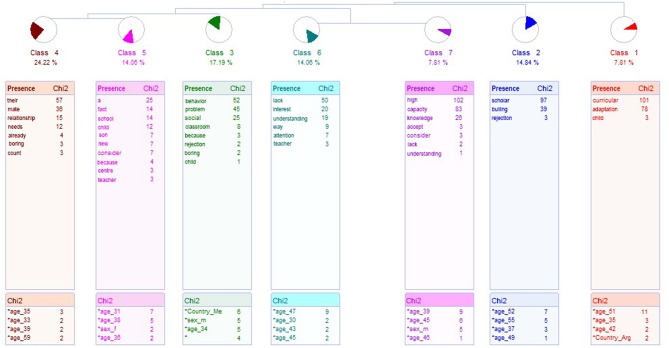
Dendrogram about children's problems with the school.

To analyze the type of problems mentioned by the children, an analysis was made with ALCESTE. It threw 7 classes, showed in the Figure 4, that allowed to classify 128 ECUs, which represents 68% of the corpus. First class connects to the rest with a hierarchical structure, connecting the second class with others. They are divided into the other two axes, one of them connects classes 6 and 7 and the second axis related to class 3 with a secondary axis in classes 4 and 5.

Class 1, Lack of Curricular Adaptation, groups 10 ECUs, which represents 7.81% of the corpus. The most significant word is *Curricular*. Examples of this class are shown below.

Unité textuelle n°28 de Khi2 = 37

no hicieron la adaptación curricular (Individu n° 27 ^*^sex_f ^*^age_35 ^*^Country_Spain)

Unité textuelle n°127 de Khi2 = 37

no se hace adaptación curricular.

*they did not do the curricular adaptation* (chi^2^ = 37)

*no curricular adaptation is made* (chi^2^ = 37)

Class 2 is called Bullying, and groups 18 ECUs, which represents 14.06% of the corpus. The most representative word is *lack*. Examples of this class are presented below.

Unité textuelle n°30 de Khi2 = 24

acoso escolar, desprecio, dejadez (Individu n° 29 ^*^sex_f ^*^age_43 ^*^Country_Spain)

Unité textuelle n°99 de Khi2 = 24

acoso escolar.

*bullying, contempt, sloppiness* (chi^2^ = 24)

*bullying* (chi^2^ = 24)

Class 3, named Behaviors Problems, include 22 ECUs (17.19% of the corpus) and the most representative word is *Behavior*. Below are two examples of phrases of this class.

Unité textuelle n°22 de Khi2 = 13

problemas de conducta (Individu n° 21 ^*^sex_f ^*^age_37 ^*^Country_Spain)

Unité textuelle n°40 de Khi2 = 13

problemas de conducta.

*Behavioral problems* (chi^2^ = 13)

*Behavioral problems* (chi^2^ = 13)

Class 4, which is connected with class 5, is named Lack of Attention to their Needs. It has 31 ECUs, that represents 24.22% of the corpus. The most representative word is *Their*. The sentence examples are shown below.

Unité textuelle n°58 de Khi2 = 11

va al mismo ritmo de sus compañeros y se aburre (Individu n° 57 ^*^sex_f ^*^age_39 ^*^Country_Spain)

Unité textuelle n°65 de Khi2 = 11

el típico ninguneo en clase y no atenderle sus necesidades.

*He/she goes at the same pace as his/her mates and gets bored* (chi^2^ = 11)

*the typical do not pay attention to him/her in the classroom and not attend to his needs* (chi^2^ = 11)

Class 5, teacher disinterest, represents 14.06% of the corpus, with 18 ECUs, being the main word *a. Two* examples are showed below.

Unité textuelle n°7 de Khi2 = 15

los profesores no me creen, me han hecho sentir como la madre tonta que cree que su hijo es mejor que los demás (Individu n° 7 ^*^sex_f ^*^age_48 ^*^Country_Spain)

Unité textuelle n°81 de Khi2 = 15

al cambiar de escuela, la orientadora considera que no es necesario hacer nada, es-cierto-que el método en el nuevo centro permite a mi hija ir a su ritmo, pero (Individu n° 79 ^*^sex_f ^*^age_38 ^*^Country_Spain

*the teachers do not believe me, they have made me feel like the foolish mother who believes that her son is better than the others* (chi^2^ = 15)

*when changing schools, the counselor considers that it is not necessary to do anything, it is true that the method in the new center allows my daughter to go at her own pace* (chi^2^ = 15)

The last axis is formed by the classes 6 and 7. Class 6, named Lack of Interest, includes 18 ECUs (14.06 % of the corpus). The most representative word is *Lack*. Examples of phrases are shown in the following paragraphs.

Unité textuelle n°12 de Khi2 = 14

falta de información, falta de atención, falta de interés (Individu n° 11 ^*^sex_f ^*^age_41 ^*^Country_Spain)

Unité textuelle n°31 de Khi2 = 14

falta de comprensión y falta de interés (Individu n° 30 ^*^sex_f ^*^age_47 ^*^Country_Spain)

*lack of information, lack of attention, lack of interest* (chi^2^ = 14)

*lack of understanding and lack of interest* (chi^2^ = 14)

Class 7 is named Unawareness of the High Abilities, represents 7.81 % of the corpus, with 10 ECUs, and the most significative word is *High*. Examples of phrases are:

Unité textuelle n°4 de Khi2 = 24

nunca han aceptado sus altas capacidades y solo ven el asperger (Individu n° 4 ^*^sex_f ^*^age_40 ^*^Country_Spain)

Unité textuelle n°51 de Khi2 = 24

falta de conocimiento en altas capacidades.

*they have never accepted their high abilities and only see the Asperger* (chi^2^ = 24)

*lack of knowledge in high abilities* (chi^2^ = 24).

## Discussion

This work deepens the understanding of parents' assessments of the educational responses that their high-ability children receive. The specific research questions of the study are (1) to examine parents' understanding of educational responses; (2) to detect which types of educational responses are implemented and what parents believe about them; and (3) to identify the problems that both parents and children have in relation to the children's schools.

Regarding research question 1, the study's analysis separates parents whose children received an educational response from those whose children did not. Clear differences appeared; for instance, the answers of the first group involved two classes, but the answer of the second group had seven classes. In both groups, one of the classes corresponded to the traditional educational response for these students. This was the most common type of response for children who did not receive an educational response that matched with their high abilities, but it was less common among those who did receive an appropriate response.

However, the parents in each group had non-traditional ideas that involved empowering their children's capacities through education that addresses emotional and social aspects.

In reference to research question 2, the first aspect that should be highlighted is that only about a quarter of the participants reported that their children received educational responses that fulfilled their needs. These results are consistent with these students' school situations, as previously noted; in particular, many fewer Spanish and Mexican school children received educational responses than would be expected: 0.33% of Spanish schoolchildren (at all levels, from preschool to the university level) and 0.7% of Mexican primary-school students. This study deals with families whose high-ability children have strong educational involvement; nevertheless, three quarters of the sample did not receive in-school educational responses.

It is also important to emphasize that this study provides a positive assessment of in-school programs. These programs are carried out in a short period after identification, and parents value them highly in terms of both the teachers and the content. Regarding the type of program, it is striking that most parents in this study reported that their children received various educational responses.

The rate of extracurricular responses was much higher than that of in-school responses, and the two types were sometimes combined. This shows that parents sought solutions that would meet their children's educational needs when the educational system did not do so. This is not surprising, as it is common for parents to create associations and to seek alternatives. There is a clear lack of attention paid to these high-ability students, in terms of both applying existing laws and providing institutional monitoring that can allow for appropriate responses for these students.

With regard to the third research question, this study identified the educational problems that the parents and their children experience. For the parents, the schools' inability to deal with their children was based on a wide range of problems, as is evident in the seven classes ALCESTE produced for this area. The problems parents have with the school, both specific for parents and for children, gathers a wide sort of problems divided in three types. Parents perceive lack of interest and knowledge about high intellectual abilities which has consequences in their children like behavioral problems or bullying. It would be necessary to study this issue more in depth this situation between parents and the school in order to avoid conflicts and improve the educational response for these children. On that sense the knowledge of the teachers on this field is essential.

Clearly, parents collect their own complaints regarding their relationship with the school in addition to complaints related to their children's problems.

The problems reported in this study include a wide range, including absence of educational response (which is a well-documented reality, as indicated in the introduction); lack of attention and consideration given to high-ability students; and even the children's disruptive behaviors, which can be partly explained by the absence of adequate educational responses. However, although some researchers have reported a high rate of bullying in this student population (e.g., Soler, 2017), bullying was not a frequent complaint among the parents in this study's sample.

One limitation of this study is the size of its sample. Although the number of participants is high, it would ideally be higher, as it covers three countries. However, given the lack of a policy that ensures the identification of all high-ability students, it is difficult to obtain samples with an adequate frame of reference for a probabilistic sampling to be carried out. Therefore, it is highly recommended for other researchers to replicate this study with a larger sample and to expand the research to other countries. For example, the study is being replicated with Portuguese-speaking parents in Portugal and Brazil. Also, it would desirable to include the perception of teachers on this type of studies, in order to analyze their perception about the educational interventions available in the schools for this population.

Finally, the use of mixed-methods research (MMR) is the strongest point of this investigation. Directly analyzing the respondents' responses without the need to restrict the survey to the usual closed-ended questions, allows for a greater understanding of exactly what the respondents think. For instance, in this study, the mixed-methods survey helped determine that the participants' expectations were far from the usual educational responses that they received.

## Ethics Statement

The University of La Laguna's Ethics Committee of Research and Animal Welfare has approved this research (registration number CEIBA2018-0300).

## Author Contributions

ER-N and MC have participated in theoretical review and method. ÁB and DV has participated in the analysis. ÁB has participated in discussion. All authors have participated in the study planning, writing and revision of the article.

### Conflict of Interest Statement

The authors declare that the research was conducted in the absence of any commercial or financial relationships that could be construed as a potential conflict of interest.

## Footnotes

^1^Ley Orgánica 2/2006, 3 de Mayo de Educación (LOE), BOE, n°106, 4 de mayo de 2006.

^2^Ley Orgánica 8/2013, de 9 de diciembre, para la mejora de la calidad educativa, BOE, n°295, 10 de diciembre de 2013.

## References

[B1] Arranz E. (2005). Family context and psychological development in early childhood: Educational implications, in Contemporary perspectives in early childhood education, families and communities, eds Saracho O. Spodeck B. (Greenwich: Information Age Publishing), 59–82.

[B2] Bauer M. (2003). Análisis de textos asistidos con programas computacionales. Subjet. Proces. Cogn. 3, 101–111.

[B3] Borges A. Rodríguez-Dorta M. Rodríguez-Naveiras E. (2016a). El programa Encuentros: La integración de los programas para progenitores en la intervención con alumnado de altas capacidades. AMAzônica. XVIII, 213–236.

[B4] Borges A. Rodríguez-Dorta M. Rodríguez-Naveiras E. Nieto I. (2016b). Buenas Prácticas en la Respuesta Educativa al Alumnado de altas Capacidades. 3° Congreso da Ordem dos Psicólogos Portugueses(Lisboa).

[B5] Borges A. Rodríguez-Naveiras E. Rodríguez-Dorta M. (2017). Perfil de Adaptación Personal y Social de Los Participantes Del Programa Integral Para Altas Capacidades (PIPAC). Comunicación presentada en el III Congreso Nacional de Psicología (Oviedo).

[B6] Bronfenbrenner U. (Ed.). (2005). Making Humans Being Human. Bioecological Perspectives on Human Development. London: Sage Publications.

[B7] Bronfrenbrenner U. Morris P. A. (1998). The ecology of developmental processes, in Handbook of Child Psychology. Vol. 1: Theoretical Models of Human Development, eds Damon W. Lerner R. M. (New York, NY: Wiley), 993–1028.

[B8] Cadenas M. Borges A. (2012). Estudio de las expectativas parentales hacia un programa de intervención. Revista de Investigación y Divulgación en Psicología y Logopedia, 2, 16–20.

[B9] Cadenas M. Borges A. (2017). The assessment of change in social interaction through observation. Acción Psicol. 14, 121–136. 10.5944/ap.14.1.16224

[B10] Castellanos Simons D. Bazán Ramírez A. Ferrari Belmont A. M. Hernández Rodríguez C. A. (2015). Apoyo familiar en escolares de alta capacidad intelectual de diferentes contextos socioeducativos. Rev. Psicol. 33, 299–332.

[B11] Colangelo N. Assouline S. (2009). Acceleration: meeting the academic and social needs of students, in The Routledge International Companion to Gifted Education, eds Balchin T. Hymer B. Matthews D. J. (London; New York, NY: Routledge; Taylor and Francis Group), 194–202.

[B12] Colangelo N. Assouline S. G. Gross M. U. (2004). A Nation Deceived: How Schools Hold Back America's Brightest Students. The Templeton National Report on Acceleration. Vol. 2. Connie Belin and Jacqueline N. Blank International Center for Gifted Education and Talent Development (NJ1).

[B13] Colangelo N. Davis G. A. (2003). Handbook of Gifted Education, 3rd Edn. Boston, MA: Ally and Boston.

[B14] Courtinat-Camps A. Massé L. de Léonardis M. Capdevielle-Mougnibas V. (2017). The heterogeneity of self-portraits of gifted students in France. Roeper Rev. 39, 24–36. 10.1080/02783193.2016.1247396

[B15] Cross T. L. Coleman L. J. (2005). School-based conception of giftedness, in Conceptions of Giftedness, eds Sternberg R. Davidson J. E. (Cambridge: Cambridge University Press), 52–63. 10.1017/CBO9780511610455.005

[B16] De Alba M. (2004). El Método ALCESTE y su aplicación al estudio de las representaciones sociales del espacio urbano: el caso DE LA CIUDAD de México, in Papers on Social Representations, 13.

[B17] Denscombe M. (2008). A research paradigm for the mixed methods approach. J. Mixed Methods Res. 2, 270–283. 10.1177/1558689808316807

[B18] Elices J. A. Palazuelos M. del Caño M. (2013). Alumnos con Altas Capacidades Intelectuales. Caracterí*sticas, Evaluación y Respuesta Educativa* Madrid: Editorial CEPE.

[B19] Gagné F. (2015). Academic talent development programs: a best practices model. Asia Pacific Educ. Rev. 16, 281–229. 10.1007/s12564-015-9366-9

[B20] Garn A. C. Jolly J. L. (2015). A model of parental achievement-oriented psychological control in academically gifted students. High Ability Stud. 26, 105–116. 10.1080/13598139.2015.1028614

[B21] Garn A. C. Matthews M. S. Jolly J. L. (2010). Parental influences on the academic motivation of gifted students: a self-determination theory perspective. Gifted Child Q. 54, 263–272. 10.1177/0016986210377657

[B22] Garn A. C. Matthews M. S. Jolly J. L. (2012). Parents' role in the academic motivation of students with gifts and talents. Psychol. Schools 49, 656–667. 10.1002/pits.21626

[B23] Geake J. G. (2009) Neuropsychological characteristics of academic and creative giftedness, in *International Handbook of Giftedness*, ed Shavinina L. V. (Dordrecht: Springer), 261–73.

[B24] Hernández D. Gutiérrez-Sánchez M. (2014). El estudio de la alta capacidad intelectual en España: análisis de la situación actual. Rev. Educ. 364, 251–272.

[B25] Hoogeveen L. (2008). Social emotional consequences of accelerating gifted students. (Doctoral thesis). Radboud Universiteit Nijmegen, Nijmegen, Netherlands.

[B26] Hoogeveen L. van Hell J. G. Verhoeven L. (2012). Social-emotional characteristics of gifted accelerated and non-accelerated students in the Netherlands. Br. J. Educ. Psychol. 82, 585–605. 10.1111/j.2044-8279.2011.02047.x 23025394

[B27] Illia L. Sonpar K. Bauer M. W. (2014). Applying co-occurrence text analysis with ALCESTE to studies of impression management. Br. J. Manag. 25,352–372. 10.1111/j.1467-8551.2012.00842.x

[B28] Jen E. (2017). Affective interventions for high-ability students from 1984-2015: a review of published studies. J. Adv. Acad. 28, 225–247. 10.1177/1932202X17715305

[B29] Jen E. Gentry M. Moon S. M. (2017). High-ability students' perspectives on an affective curriculum in a diverse, university-based summer residential enrichment program. Gifted Child Q. 61, 328–342. 10.1177/0016986217722839

[B30] Jiménez J. Artiles C. Ramírez G. Álvarez J. (2006). Evaluación de los efectos de la aceleración en alumnos con alta capacidad intelectual en la Comunidad Autónoma de Canarias. Infancia y Aprendizaje 29, 51–64. 10.1174/021037006775380975

[B31] Johnson R. B. Onwuegbuzie A. J. (2004). Mixed methods research: a research paradigm whose time hascome. Educ. Res. 33, 14–26. 10.3102/0013189X033007014

[B32] Kim M. (2016). A meta-analysis of the effects of enrichment programs on gifted students. Gifted Child Q. 60, 102–116. 10.1177/0016986216630607

[B33] Kulik J. A. (2004). Meta-analytic studies of acceleration, in A Nation Deceived: How Schools Hold Back America's Brightest Students, Vol. II, eds Colangelo N. Assouline S. G. Gross M. U. M. (Iowa City, IA: Belin and Blank International Center for Gifted Education and Talent Development; University of Iowa), 13–22.

[B34] López Andrada B. Beltrán M. T. López B. Chicharro D. (2000). CIDE: Alumnos Precoces, Superdotados y de Altas Capacidades. Madrid: Centro de investigación y documentación educativa (CIDE).

[B35] Matthews D. J. Yun Dai D. (2014). Gifted education: changing conceptions, emphases and practice. Int. Stud. Sociol. Educ. 24, 335–353. 10.1080/09620214.2014.979578

[B36] Miller R. Gentry M. (2010). Developing talents among high-potential students from low-income families in an out-of-school enrichment program. J. Adv. Acad. 21, 594–627. 10.1177/1932202X1002100403

[B37] Ministerio de Educación y Formación Profesional (2017). Enseñanzas no Universitarias. Necesidades de Apoyo Educativo. Curso 2016-207. Retrieved from http://estadisticas.mecd.gob.es/EducaDynPx/educabase/index.htm?type=pcaxisandpath=/Educacion/Alumnado/Apoyo/Curso1617/AltasCapacidadesandfile=pcaxisandl=s0

[B38] Ministerio de Educación 000F3;n. Pacto Federal Educativo. Buenos Aires: Departamento Impresiones.

[B39] Mönks F. J. (1992). Developmentof gifted children: the issue of identification and programming, in Talent for the Future, eds Mönks F. J. Peters W.A. M. (Maastricht: Van Gorcum), 191–202.

[B40] Parrello S. Osorio-Guzmán M. (2013) Reconstrucción narrativa de una experiencia de hospitalización. Rev. Costarricense Psicol. 32, 177–192.

[B41] Pérez L. (2006). Alumnos Con Capacidad Superior. Experiencias de intervención educativa. Madrid: Síntesis.

[B42] Pérez L. (2012). Programas educativos para alumnos con alta capacidad: sistemas de enriquecimiento, in Alumnos Superdotados y Talentosos. Identificación, Evaluación e Intervención. Una Perspectiva Para Docentes, eds Valadez D. V Betancourt J. Zavala M. A. (Mexico: Manual Moderno), 155–187.

[B43] Reinert M. (2003). Alceste Users' Manual. Touluse: Image.

[B44] Rodríguez-Dorta M. Rodríguez-Naveiras E. Borges del Rosal A. (2017). Los programas para progenitores: evaluación de los dos niveles del Programa Encuentros, in Comunicación Presentada as III Jornadas Internacionales Sobre Panorámica de Intervención en Altas Capacidades Intelectuales (La Laguna).

[B45] Rodríguez-Naveiras E. Borges A. (2016). Evaluación formativa y sumativa del Programa Integral para Altas Capacidades (PIPAC), in Programas de Intervención Para Niños Con Altas Capacidades y su Evaluación Coords, eds Valadez D. López G. Borges A. Betancourt J. Zambrano y R. (Mexico: Manual Moderno), 49–65.

[B46] Sastre-Riba S. (2008). Niños con altas capacidades y su funcionamiento cognitivo diferencial. Rev. Neurol. 46, 11–16. 10.33588/rn.46S01.2008008 18302114

[B47] Sastre-Riba S. Domenech M. (1999). La identificación diferencial de la superdotación y el talento. Faisca 7, 23–49.

[B48] Sastre-Riba S. Ortiz T. (2018). Neurofuncionalidad ejecutiva: estudio comparativo en las altas capacidades. Rev. Neurol. 66, 51–56. 10.33588/rn.66S01.2018026 29516453

[B49] Sastre-Riba S. Viana-Sanz L. (2016). Funciones ejecutivas y capacidad intelectual. Rev. Neurol. 62, 65–71. 10.33588/rn.62S01.2016025 26922961

[B50] SEP (2006). Propuesta Nacional de Intervención: Atención Educativa a Alumnos y Alumnas con Aptitudes Sobresalientes. Mexico: Secretaria de Educación Pública.

[B51] SEP (2018). Principales Cifras del Sistema Educativo Nacional. Mexico: Secretaria de Educación Pública.

[B52] Soler J. (2017). Bullying y Alumnos catalanes con Altas Capacidades. Asociación NACE. Retrieved from: https://www.noalacoso.org/informe-bullying-aacc-cataluna/

[B53] Southern W. T. Jones E. D. (2004). Types of acceleration: Dimensions and issues, in A Nation Deceived: How Schools Hold Back America's Brightest Students, Vol. II, eds Colangelo N. Assouline S. G. Gross M. U. M. (Washington, DC: National Association for Gifted Children), 5–12.

[B54] Subotnik R. F. Olszewski-Kubilius P. Worrell F. C. (2011). Rethinking giftedness and gifted education: a proposed direction forward based on psychological science. Psychol. Sci. Public Interest 12, 3–54. 10.1177/1529100611418056 26168418

[B55] Tannenbaum A. J. (1986). Giftedness: a psychosocial approach, in Conceptions of Giftedness, eds Sternberg R. J. Davidson J. E. (New York, NY: Cambridge University Press), 21–52.

[B56] Torres L. Maia E. (2009). Percepción de las madres acerca del contenido de la información del diagnóstico del Síndrome de Down. Rev. Chilena Pediatr. 80, 39–47 10.4067/S0370-41062009000100005

[B57] Tourón J. (2012). El agrupamiento por capacidad. El caso de los alumnos más capaces. In VVAA Liber Amicorum en honor del profesor Arturo de la Orden, Madrid.

[B58] Van Tassel-Baska J. (2013). Curriculum for the gifted. A commitment to excellence. Gifted Child Today 36, 213–240. 10.1177/1076217513487351

[B59] Verche E. Pérez J. Borges A. Galeano D. Serrano A. (2018). Diferencias en flexibilidad cognitiva y creatividad entre alumnado de altas capacidades y un grupo control, in Comunicación presentada en el V Congreso Internacional en Contextos Psicológicos, Educativos y de la Salud (Madrid).

[B60] Villate A. De Leonardis M. (2012). “Qui suis je?” Quelques spécificités du discours sur soi à l'adolescence chez les sujets à haut potentiel intellectuel. Neuropsychiatrie de l'Enfance et de l'Adolescence 60, 101–107. 10.1016/j.neurenf.2011.09.002

[B61] Weber C. L. Stanley L. (2012). Educating parents of gifted children: designing effective workshops for changing parent perceptions. Gifted Child Today 35, 128–136. 10.1177/1076217512437734

[B62] Zambrano R. (2017). El papel del profesorado de escuelas primarias en la propuesta nacional Mexicana de atención a alumnos con aptitudes sobresalientes (Doctoral thesis). La Laguna: Universidad de La Laguna.

[B63] Ziegler A. (2005). The actiotope model of giftedness, in Conceptions of Giftedness, eds Sternberg R. J. Davidson J. E. (New York, NY: Cambridge University Press), 411–436.

[B64] Ziegler A. Phillipson S. N. (2012). Towards a systemic theory of gifted education. High Ability Stud. 23, 3–30. 10.1080/13598139.2012.679085

